# Response Dynamics in an Olivocerebellar Spiking Neural Network With Non-linear Neuron Properties

**DOI:** 10.3389/fncom.2019.00068

**Published:** 2019-10-01

**Authors:** Alice Geminiani, Alessandra Pedrocchi, Egidio D’Angelo, Claudia Casellato

**Affiliations:** ^1^Department of Brain and Behavioral Sciences, University of Pavia, Pavia, Italy; ^2^NEARLab, Department of Electronics, Information and Bioengineering, Politecnico di Milano, Milan, Italy; ^3^IRCCS Mondino Foundation, Pavia, Italy

**Keywords:** olivocerebellar circuit, spiking neural network (SNN), point neuron, non-linear neuronal dynamics, eyeblink response

## Abstract

Sensorimotor signals are integrated and processed by the cerebellar circuit to predict accurate control of actions. In order to investigate how single neuron dynamics and geometrical modular connectivity affect cerebellar processing, we have built an olivocerebellar Spiking Neural Network (SNN) based on a novel simplification algorithm for single point models (Extended Generalized Leaky Integrate and Fire, EGLIF) capturing essential non-linear neuronal dynamics (e.g., pacemaking, bursting, adaptation, oscillation and resonance). EGLIF models specifically tuned for each neuron type were embedded into an olivocerebellar scaffold reproducing realistic spatial organization and physiological convergence and divergence ratios of connections. In order to emulate the circuit involved in an eye blink response to two associated stimuli, we modeled two adjacent olivocerebellar microcomplexes with a common mossy fiber input but different climbing fiber inputs (either on or off). EGLIF-SNN model simulations revealed the emergence of fundamental response properties in Purkinje cells (burst-pause) and deep nuclei cells (pause-burst) similar to those reported *in vivo*. The expression of these properties depended on the specific activation of climbing fibers in the microcomplexes and did not emerge with scaffold models using simplified point neurons. This result supports the importance of embedding SNNs with realistic neuronal dynamics and appropriate connectivity and anticipates the scale-up of EGLIF-SNN and the embedding of plasticity rules required to investigate cerebellar functioning at multiple scales.

## Introduction

A broad set of experimental observations has suggested that cerebellar circuit functioning relies on a number of detailed features distributed over multiple scales. Single neuron properties along with an organized modular connectivity shape population-specific spiking patterns and spatio-temporal network dynamics, which in turn determine the relationship between input stimuli and responses. The precise encoding of spatio-temporal features into the output (which is in motor domain) corresponds to the cerebellar contribution in sensorimotor tasks (Llinas and Negrello, 2011; Llinás, 2014; D’Angelo, 2018). Indeed, together with synaptic plasticity, single neuron electroresponsiveness and network connectivity affect motor learning and alterations of these elements can significantly compromise movement adaptation (Peter et al., 2016).

At the cerebellar input, the Granular layer is thought to act as a spatio-temporal filter of sensory inputs (Marr, 1969). This operation has been related to specific properties of Golgi cells (GoCs) and Granule cells (GrCs), such as oscillatory and resonant dynamics, along with the arrangement of microcircuit connectivity, which includes recurrent GoC-GrC inhibitory loops and GoC local networks (D’Angelo et al., 2013; Gandolfi et al., 2013). The GoCs contribute to process sensory signals coming from Mossy Fibers (MFs) by shaping the activity of GrCs. GrC signals converge to the Molecular and Purkinje cell layers through Ascending Axons (AAs) and Parallel Fibers (PFs), with a very precise geometrical organization. Purkinje cells (PCs) are the final integrators of the cerebellar cortex, inhibiting the cerebellar output that drives motor responses (Heiney et al., 2014). *In vivo*, intrinsic simple spikes of PCs are modulated by excitation from GrCs and inhibition from Molecular Layer Interneurons (MLIs). Moreover, inputs from Inferior Olive (IO), through Climbing Fibers (CFs), elicit PC complex spikes (Davie et al., 2008). Deep Cerebellar Nuclei cells (DCNs) are the only output of the cerebellar circuit, projecting centrally to multiple brain areas, and peripherally to the motor pathways. Integrating the inputs from the cerebellar cortex and MFs, DCNs can modify their spontaneous firing and generate pauses and bursts. Burst-pauses in PCs and pause-bursts in DCN cells are thought to be essential to finely tune the motor responses (Shadmehr, 2017). DCNs also continuously control learning processes through inhibitory feedback loops to the IO (De Zeeuw et al., 2011). The PC-DCN-IO loop connections are organized to form microcomplexes: CFs from IO sub-regions project to different sagittal stripes of PCs, which in turn receive signals from subvolumes of the granular layer and of the molecular layer (i.e., microzones); then, PCs of a microcomplex target the corresponding nuclear regions reached by the same CFs (Llinas and Negrello, 2011; Ruigrok, 2011; D’Angelo et al., 2013). On the other hand, GrCs project in the medio-lateral direction by PFs (Uusisaari and de Schutter, 2011), carrying the same signals transversally to multiple microcomplexes. The result is a modular geometrically-organized architecture, where each microcomplex integrates sensorimotor information from different sources and emits spike patterns that, in turn, correlate with specific aspects of behavior (Zhou et al., 2014; Powell et al., 2015).

In this scenario, single neuron properties and cerebellar connectivity are sufficiently well characterized and can be simplified to simulate behavioral tasks using bioinspired cerebellar models (Yamazaki and Igarashi, 2013; Casellato et al., 2014; Antonietti et al., 2016). However, the key causal relationships across scales, i.e., from neuron properties to network dynamics and finally to behavior, are still unclear. To what extent do intrinsic excitability and synaptic inputs contribute to the spiking patterns of PCs and DCN cells during a behavioral task? How do complex firing patterns emerge in cascade within the network?

Here, we have reconstructed and simulated an olivocerebellar microcircuit by integrating monocompartmental neurons with complex electroresponsiveness into the geometrically-organized connectivity of a spiking neural network (SNN). The simulations provide the network with sensory-like stimulation patterns and monitor the microcircuit responses. Such a computational tool compromises between biological plausibility and computational load, allowing a multiscale investigation of the cerebellar network. This is achieved by integrating two main aspects. The first one is the Extended-Generalized Leaky Integrate and Fire (EGLIF) point neuron that maintains salient electrophysiological features – autorhythm, bursts, adaptation, oscillations and resonance – by using just a few state variables (Geminiani et al., 2018b). The EGLIF proved capable to reproduce the rich set of firing patterns of the main olivocerebellar neurons: GoCs, GrCs, PCs, MLIs, DCNs, and IO (Geminiani et al., 2019). The second aspect is network geometry derived from a cerebellar scaffold model, which reproduces the physiological convergence and divergence ratios of connections with a realistic spatial organization (Casali et al., 2019). Here, EGLIF neurons are here evaluated within the whole SNN, where positioning and connectivity of each neuron type are based on their morphology and density within the cerebellar microcircuit (Casali et al., 2019). Therefore, the EGLIF-SNN is exploited to investigate how single neuron properties and network architecture allow the emergence of spatio-temporal dynamic properties, such as burst-pause in PCs and pause-burst in DCN cells. In particular, the EGLIF-SNN is tested by using input patterns encoding two types of sensory signals, whose timing association elicits an eyeblink motor response with multiple afferent pathways specifically activating interconnected microcomplexes (De Zeeuw et al., 2011). The simulations using EGLIF-SNN have been compared to others using simple LIF neurons, in order to understand the impact of single neuron dynamics on network functioning and signal encoding. These results provide a critical assessment of the role of microcircuit properties needed for future closed-loop simulations of cerebellum-driven learning tasks (D’Angelo et al., 2016).

## Materials and Methods

### Reconstruction of the Olivocerebellar Network

**FIGURE 1:**
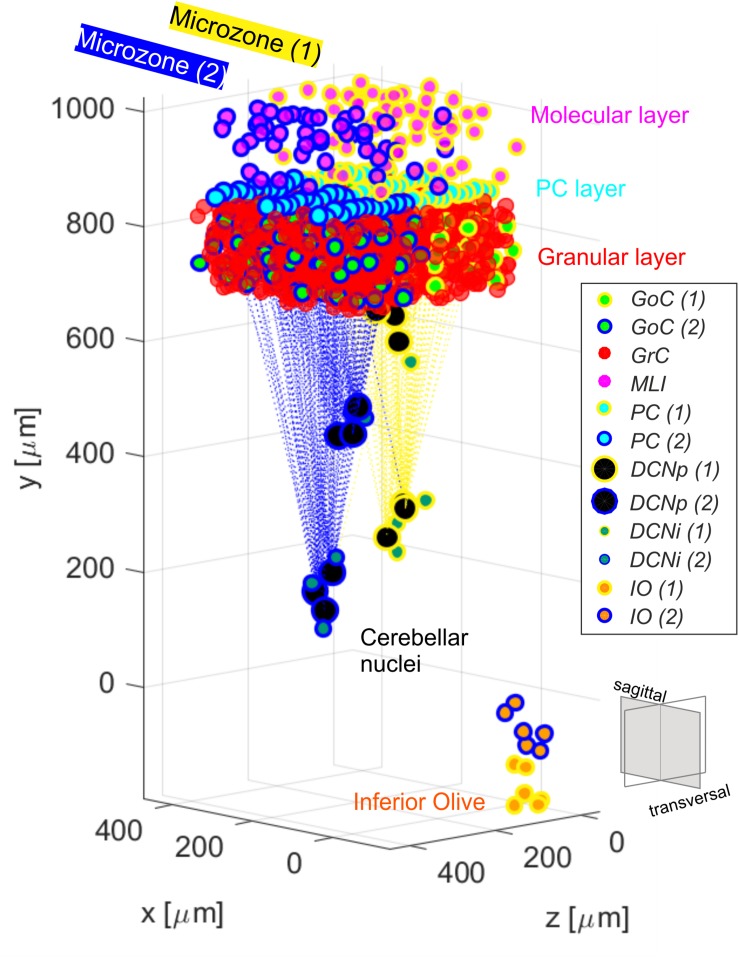
Olivocerebellar scaffold with neurons placed in the selected volume, including two cortical microzones (granular, molecular and PC layers) with their corresponding nuclear and olivary cells. Connections between PCs from each microzone and the corresponding target cells in the cerebellar nuclei are highlighted. The two microcomplexes are labeled in yellow (1) and blue (2). Granular and molecular layer cells are subsampled and GrC in the two microzones are not differently labeled for figure readability.

To evaluate the role of single neuron electrophysiology and, at the same time, of geometrical and statistical connectivity, a SNN was developed, reproducing an olivocerebellar volume. The reconstructed volume included 96′767 neurons and 4′151′182 total synapses and represented a portion of two cerebellar microcomplexes with the corresponding olivary nuclei (Figure 1). The SNN was built based on the cerebellar scaffold developed in Casali et al. (2019). In this scaffold, neurons were placed in the selected volume based on known cell densities from neurophysiology and geometric features. Then, they were connected according to connectivity rules based on proximity of neuronal processes (pre-synaptic axon span extension and post-synaptic dendritic field extension) and on statistical convergence/divergence ratios (Casali et al., 2019). The starting network version was made up of cells distributed in a multi-layered volume including the Molecular, Purkinje and Granular layers of the cerebellar cortex – 400 × 330 × 400 μm^3^, and the underlying cerebellar nuclei – 200 × 600 × 200 μm^3^ (Table 1). The thickness (along y-direction) was fixed based on neurophysiology (330 μm for cortex + 600 μm for nuclei), while the other two sizes (x and z) were flexible, and there defined in order to have a complete exemplificative reconstruction, able to include all the elements in a functional representative module. Here, we subdivided the scaffold cortex into two sub-volumes, by a parasagittal plane, so obtaining two microzones with a transversal length of 200 μm each (along *z*-axis). Consequently, we reorganized the PC-DCN connections to be confined within the same subvolumes, with a neurophysiological crosstalk. This way, two adjacent microcomplex volumes were reconstructed (Uusisaari and de Schutter, 2011). Then, we added an olivary volume of 100 × 200 × 40 μm^3^ chosen to maintain the ratio between the cerebellar cortical volume and the olivary one measured in mice, i.e., ∼ 66–68:1 (Lein et al., 2007). Based on IO neuron density (i.e., ∼ 15′172 cells/mm^3^), we positioned 12 cells in the olivary scaffold volume (Zanjani et al., 2004). The neurons were placed using self-avoiding bounded random walk procedure. For each olivocerebellar microcomplex, six IO neurons were included.

**TABLE 1:** Neuron types and numbers in the olivocerebellar scaffold.

**Neuron type**	**Number of neurons**
*MF*	7073
*GoC*	219
*GrC*	88164
*MLI*	1206
*PC*	69
*DCNp*	12
*DCNi*	12
*IO*	12

In the cerebellar nuclei, we considered two types of neurons: non-GABAergic DCNs, which are the principal large neurons projecting outside the cerebellum in an excitatory way (DCNp), and GABAergic interneurons (DCNi), which send inhibitory feedback signals to IO. For each DCNp, already present in the previous scaffold release (Casali et al., 2019), we added one DCNi, positioned around the corresponding DCNp, at a random distance *d* in the range between *d*_1_ (minimum to avoid somata overlap) and *d*_2_ (maximum in order to have a DCNi as a satellite of a specific DCNp, i.e., closer to that DCNp than to the other DCNp neurons):

d=1r+D⁢C⁢N⁢prD⁢C⁢N⁢i

d=2mean_dist/4-r-D⁢C⁢N⁢prD⁢C⁢N⁢i

where:

r,D⁢C⁢N⁢pr=D⁢C⁢N⁢iradiusofDCNneurons′somata;

*mean_dist* = mean pairwise distance between DCNp in the scaffold (Casali et al., 2019).

Connections to and from IO were organized to mimic the geometry of microcomplexes. IO and DCN neurons were divided into two clusters based on their position and connected to PCs in homologous microzones. This topological segregation was maintained also in connecting IO to DCNp, and DCNi to IO cells.

Furthermore, also the connections from IO to MLIs were introduced following the microcomplex correspondence (Szapiro and Barbour, 2007; Jörntell et al., 2010). The resulting convergence/divergence values of the connections within the entire olivocerebellar scaffold are reported in Table 2.

**TABLE 2:** Olivocerebellar scaffold connections with convergence/divergence ratios (reported as mean ± Standard Deviation, SD) and corresponding synaptic parameters.

	**Convergence (mean ± SD)**	**Divergence (mean ± SD)**	**Weight [nS]**	**Delay [ms]**	**τ _α_ [ms]**	**Connection type**
*MF-GrC*	4	50 ± 22	0.15	4.0	5.8 (Prestori et al., 2008)	*exc*
*MF-GoC*	65 ± 27	2 ± 1	1.5	4.0	0.23 (Kanichay and Silver, 2008)	*exc*
*GoC-GrC*	2 ± 1	624 ± 267	0.6	2.0	13.6 (Mapelli et al., 2009)	*inh*
*GoC-GoC*	34 ± 8	34 ± 9	0.3	1.0	10	*inh*
*AA-GoC*	360 ± 81	1	1.2	2.0	0.5 (Kanichay and Silver, 2008)	*exc*
*PF-GoC*	1600	4 ± 2	0.05	5.0	0.5 (Kanichay and Silver, 2008)	*exc*
*MLI-MLI*	4 ± 2	4	0.2	1.0	2	*inh*
*PF-MLI*	1004 ± 221 (BC) 1021 ± 221 (SC)	12 ± 4 (BC) 12 ± 5 (SC)	0.015	5.0	0.64	*exc*
*MLI-PC*	20	3 ± 1	0.3	4.0 (BC) 5.0 (SC)	2.8 (He et al., 2015)	*inh*
*AA-PC*	249 ± 13	1	0.7	2.0	1.1 (Miyazaki et al., 2004)	*exc*
*PF-PC*	28401 ± 776	23 ± 3	0.007	5.0	1.1 (Miyazaki et al., 2004)	*exc*
*PC-DCNp*	26 ± 2	5 ± 1	0.4	4.0	0.7 (Uusisaari and Knöpfel, 2008)	*inh*
*PC-DCNi*	26 ± 4	5 ± 1	0.12	4.0	1.14 (Uusisaari and Knöpfel, 2008)	*inh*
*MF-DCNp*	147	1	0.05	4.0	1 (Wu and Raman, 2017)	*exc*
*CF-PC*	1	6 ± 1 (min = 4; max = 8)	350.0	4.0 (Llinás, 2014)	0.4 (Miyazaki et al., 2004)	*exc*
*CF-MLI*	3 ± 1	115 ± 23	1.0	70.0 ± 10.0 (De Zeeuw et al., 2011)	1.2 (Szapiro and Barbour, 2007)	*exc*
*IO-DCNp*	6	6	0.1	4.0 (Hoebeek et al., 2010)	1 (Wu and Raman, 2017)	*exc*
*IO-DCNi*	6	6	0.2	5.0	3.64 (Uusisaari and Knöpfel, 2008)	*exc*
*DCNi-IO*	6	6	3.0	20.0 (Best and Regehr, 2009)	60.0 (Best and Regehr, 2009)	*inh*

Single neurons in the SNN were modeled as EGLIF, able to reproduce the full set of spiking patterns of cerebellar neurons (Geminiani et al., 2018b). In details, a cell-specific parameter set was applied to meet the electroresponsive phenotype of each olivocerebellar neuron (e.g., GoC: autorhythm, adaptation, rebound bursting, phase reset, subthreshold oscillations, resonance; GrC: subthreshold oscillations and resonance; PC: autorhythm and bursting; DCN: autorhythm, adaptation and rebound bursting; IO: subthreshold oscillations, rebound spiking, phase reset), as optimized in Geminiani et al. (2019) (Supplementary Material). Only the firing irregularity parameters were modified with respect to Geminiani et al. (2019), to account during network simulations for higher noise components that are absent during *in vitro* experiments (Supplementary  Material). As a result, we obtained physiological Coefficient of Variation of Inter-Spike Intervals (CV_ISI_) and average firing frequency (*f*_*tonic*_) observed *in vivo* (Ten Brinke et al., 2017; Boele et al., 2018). Specifically, PCs showed *f*_*tonic*_ = 85 Hz and CV_ISI_ = 0.2, and DCNp, *f*_*tonic*_ = 65 Hz and CV_ISI_ = 0.2.

Then, the same reconstructed circuit was populated by basic LIF neurons (LIF-SNN). The passive membrane parameters were set equal for EGLIF and LIF neurons, specific for each neuron type (Supplementary Material). The intrinsic current generating spontaneous firing was tuned in the LIF neurons using trial and error, to obtain the same desired autorhythm rates.

Synaptic transmission was regulated by alpha-shaped conductance-based synapses, where reversal potentials were set to 0 mV for all excitatory synapses and −80 mV for inhibitory synapses (Cavallari et al., 2014). Multiple synapses on the same post-synaptic neuron were introduced in order to modulate the impact of different pre-synaptic populations, by using *ad hoc* synaptic parameters. The time constants of the conductance functions (τ_α_) and the synaptic delays were defined based on scaffold values (Casali et al., 2019) and literature data (Table 2). Synaptic weights were set through trial and error in order to generate reference firing rates of each neural population, during baseline state of the network, i.e., without external stimuli. In setting those synaptic weights, qualitative and comparative information were taken as constraints, e.g., the robust connections from IOs to PCs through CFs, and the stronger effect of GrCs on the post-synaptic neuron when the connection is through AAs than through PFs (Casali et al., 2019). Since the non-synaptic “spill-over” interaction between CFs and MLIs (Szapiro and Barbour, 2007; Jörntell et al., 2010), delay values of CF-MLI connections were set not all equal, but randomly chosen within a normal distribution to represent the slow and gradual neurotransmitter release. Short 1 ms delays (corresponding to the simulation resolution) were used in the interneuron inhibitory subnetworks (GoCs-GoCs and MLIs-MLIs) to mimic gap junctions (Hahne et al., 2015). The same synaptic delays and weights were used in both EGLIF-SNN and LIF-SNN, to ensure that the response differences between the two models could be ascribed unequivocally to different single neuron dynamics.

### Network Stimulation Protocol and Data Analysis

The reconstructed olivocerebellar network with optimized cell-specific neuron models (Geminiani et al., 2019) was then simulated in PyNEST (Diesmann and Gewaltig, 2002; Eppler et al., 2009). The emergent spatio-temporal dynamics was analyzed, such as the responses of all neuron populations to sensory signals involving different input pathways. To understand the impact of single neuron dynamics in emerging properties at network and signal encoding level, the same simulation protocols were applied in the two network models, EGLIF-SNN and LIF-SNN.

The chosen input signals mimic those used in EyeBlink classical conditioning (EBCC), a well-known cerebellum-driven task, commonly used to investigate cerebellar learning and the underlying circuit mechanisms (Jirenhed et al., 2007). Recruiting different sensory pathways, the input signals during EBCC are usually a continuous light signal (a LED) and a time-locked short air puff stimulation on the eye. On the other hand, the motor response is an eye closure. Our model focused on the beginning of this task, when timing associative learning has not occurred yet, and only the second stimulus is supposed to generate an attention-triggered motor response. Within our SNN, the light stimulus was encoded as a 40 Hz Poisson process conveyed through a wide MF bundle investing both microcomplexes. Moreover, transversal PF projections from the Granular layer and MF collaterals to DCN cells allow the signals to travel across adjacent microcomplexes (Kalmbach et al., 2010). The air puff was a 500 Hz burst conveyed to CFs belonging specifically to one microcomplex (Ten Brinke et al., 2015, 2017). The output motor response was decoded from the net spiking activity of DCNp neurons.

The network testing protocol included a first 1-s baseline phase with a 4 Hz Poisson process to MFs. This baseline input simulated the typical *in vivo* background noise (Rancz et al., 2007). Afterward, a 40-Hz MF spike train (associated to LED light) started, lasting 260 ms. It co-terminated with the 500-Hz CF burst (associated to air puff) which lasted 10 ms. A final 500-ms phase was added after this stimulation pair, to evaluate the capability of the network to return to baseline rest condition (Ten Brinke et al., 2015, 2017).

The input spike train activated a MF bundle in the scaffold network, specifically a cylinder with a basis radius of 150 μm at the center of the transversal *x*–*z* plane, and a height of 150 μm thus including the whole granular layer thickness. This activation pattern was chosen based on the experimental observation that cerebellar activation is region specific and topographically organized, with MFs activating in bundles eliciting local responses (Morissette and Bower, 1996; Diwakar et al., 2011). In addition, this pattern allowed to avoid edge effects due to truncated connectivity close to the borders. As a result, about 80% of glomeruli received the afferent input.

To avoid unnatural synchronization of populations’ initial spikes, the membrane potential of each neuron was initialized to a random value between the population-specific resting potential and threshold potential, in both EGLIF-SNN and LIF-SNN.

Raster plots of example neurons were used to visualize single neuron responses, while the network activity was represented as PeriStimulus time histograms (PSTH) with time bin = 5 ms, for each neural population at rest and during the imposed stimulation patterns.

PC and DCNp populations represented the convergence stages of both input stimuli pathways. Therefore, the instantaneous firing rates of PC and DCNp neurons in the first microcomplex (the one receiving both MF input and CF burst) were computed as the convolution between the neuron spiking patterns and a gaussian sliding window of 5 ms and 10 ms, respectively (Dayan and Abbott, 2001). To evaluate the difference in the responses between EGLIF-SNN and LIF-SNN, for each PC and DCNp neuron, we measured the activity change – response *speed* – following the second stimulus (i.e., CF burst):

$$s\,p\,e\,e\,d_{i}=\frac{m\,a\,x\,\_\,r\,a\,t\,e_{i}-m\,i\,n\,\_\,r\,a\,t\,e_{i}}{\Delta \,t}\;f\,o\,r\,e\,a\,c\,h\,n\,e\,u\,r\,o\,n\,i,$$

being $m\,a\,x\,\_\,r\,a\,t\,e_{i}$ and *min*_*rate*_*i*_, the maximum and minimum firing rate of the *i-th* neuron within the 100-ms interval starting 5 ms after the CF burst onset, and Δ*t* the time interval between them.

Finally, the resulting motor response was computed from DCNp activity: the spiking pattern of each microcomplex was first decoded using an update and decay rule (update constant: 1.0; decay time constant: 10 ms) and then filtered with a moving average filter using a 50-sample window. The final eyeblink response was computed from the net decoded activity of both microcomplexes.

## Results

The olivocerebellar SNN was organized into two cortical *microzones*, distinguished by their connections from CFs while sharing information from the granular layer (Voogd and Glickstein, 1998). The two microzones, differentially connected to DCN and IO, formed two distinct *microcomplexes* (Ito, 1984; Figure 1). The olivocerebellar SNN was able to encode different inputs into output spike patterns. We have analyzed in detail the response to spike trains imitating EBCC-like sensory inputs. The comparison between the EGLIF-SNN and LIF-SNN allowed to identify the contribution of non-linear single neuron properties to ensemble network dynamics.

**FIGURE 2:**
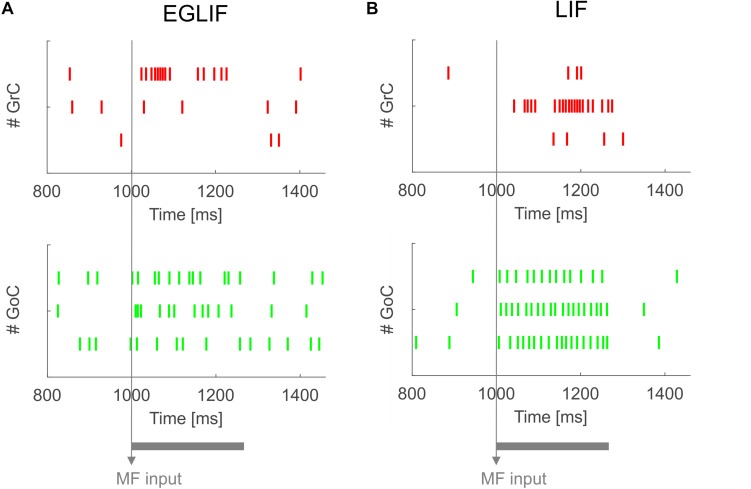
Raster plots of three examplar GrC and GoC neurons from EGLIF SNN **(A)** and LIF-SNN **(B)** simulations. The stimulation paradigm (MF input) is indicated.

**FIGURE 3:**
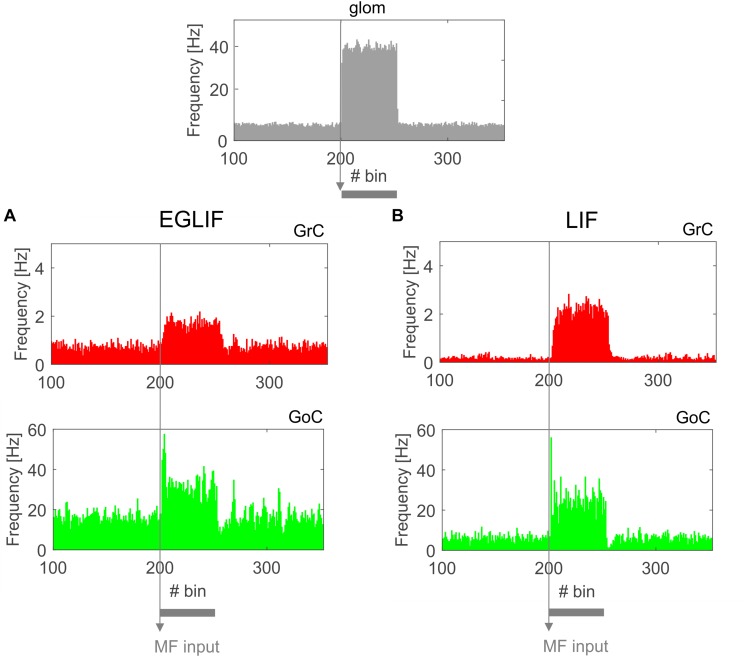
PSTH of all Granular layer neurons in EGLIF-SNN **(A)** and LIF-SNN **(B)**. In both cases, stimulation of a MF bundle common to both microcomplexes (top panel) causes the mean firing rate of granule cells and Golgi cells to increase. Note the similar patterns of neuronal activity in the two networks. The absolute values of firing rates are within physiological ranges *in vivo*. Each PSTH bin is 5 ms long.

**FIGURE 4:**
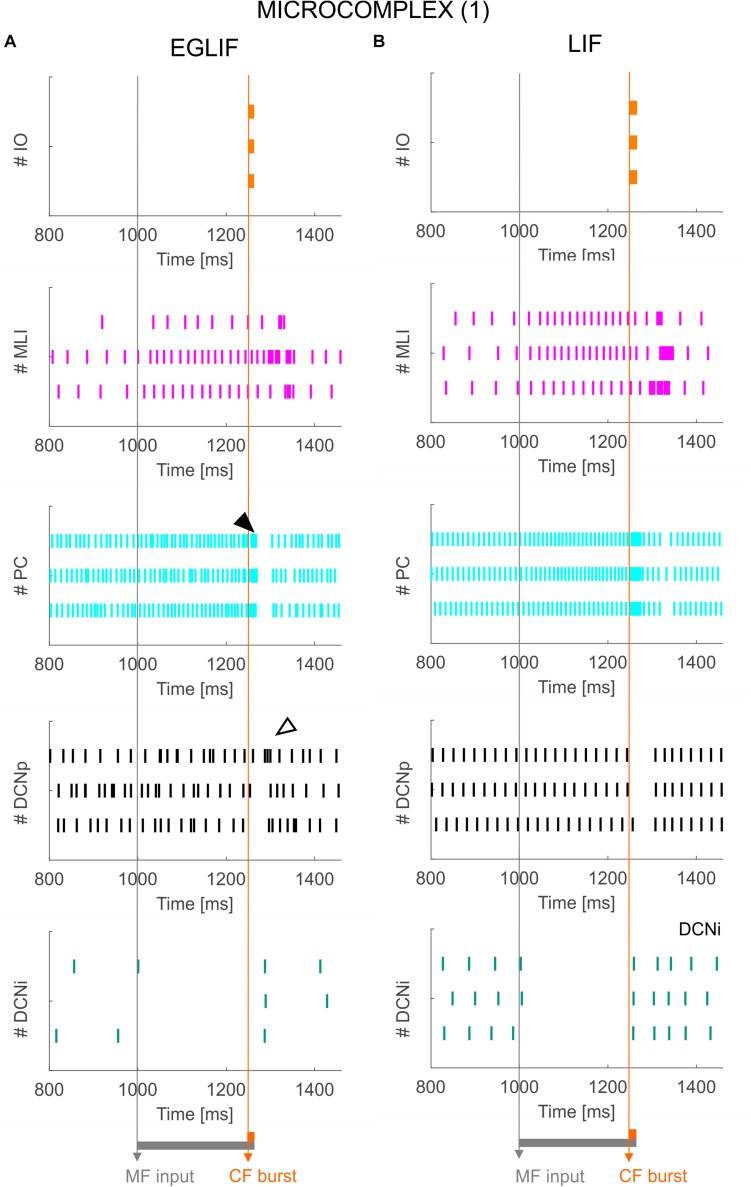
Raster plots of three example IO, MLI, PC, DCNp and DCNi neurons in the first microcomplex, from EGLIF-SNN **(A)** and LIF-SNN **(B)** simulations. The stimulation paradigm (MF input and CF burst) is indicated.

**FIGURE 5:**
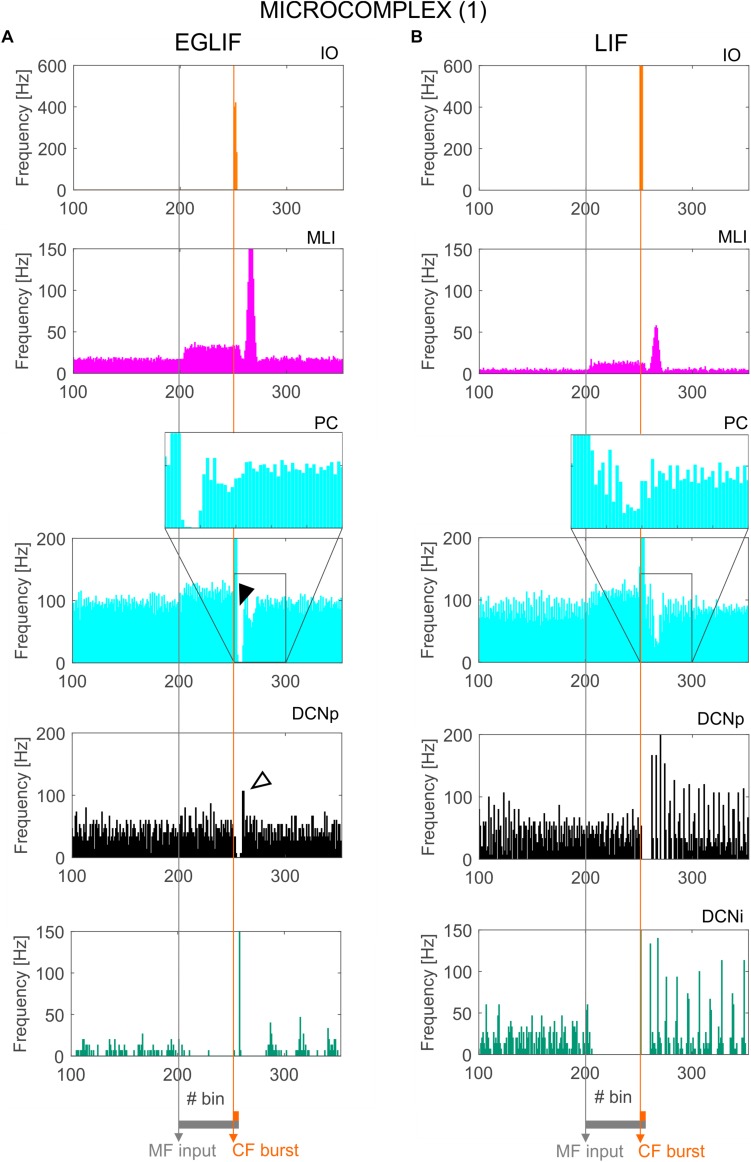
PSTH of IO, MLI, PC and DCN neurons in microcomplex (1) in EGLIF-SNN **(A)** and LIF-SNN **(B).** The first stimulus (MF input) increases the firing rate in MLI, PC and DCNp neurons during the 260 ms interval, while DCNi cells that do not receive MF inputs, get inhibited by the increased PC firing. The air puff is encoded as a burst from CFs. MLIs receive the CF stimulus through the IO pathway causing a delayed protracted increase in firing rate about 70 ms after the stimulus, due to neurotransmitter spillover from CFs. At PC level, CF stimulation results in a complex spike (burst-pause, black arrow) causing a pause-burst in DCN neurons (white arrow). Note that these dynamic behaviors are observed only in the EGLIF-SNN due to the complex intrinsic dynamics of EGLIF neuron models. In LIF-SNN, the PC burst caused by CF input is not followed by the pause, while in DCNp neurons the pause due to PC complex spike inhibition is followed by a synchronous restart of firing (causing the increased instantaneous frequency) without any rebound burst. Note that the lower irregularity of firing in LIF-SNN simulations resulted in apparent higher firing rates, due to non-physiological synchronization of population spikes. Each PSTH bin is 5 ms long.

**FIGURE 6:**
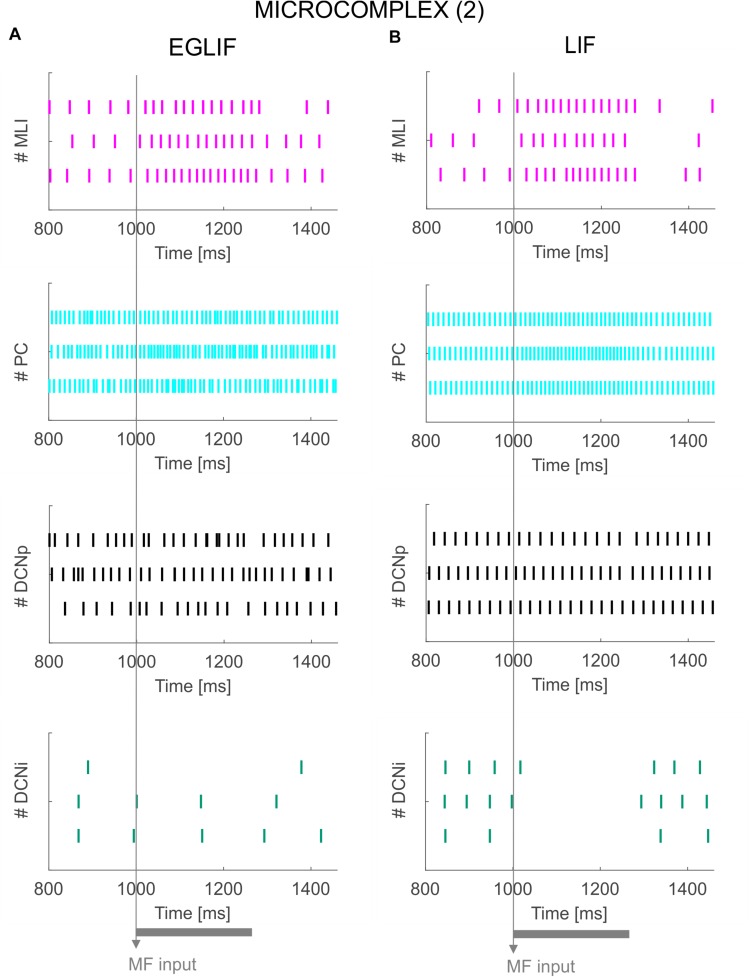
Raster plots of three example MLI, PC, DCNp and DCNi neurons in the second microcomplex, from EGLIF-SNN **(A)** and LIF-SNN **(B)** simulations. The stimulation paradigm (MF input) is indicated.

**FIGURE 7:**
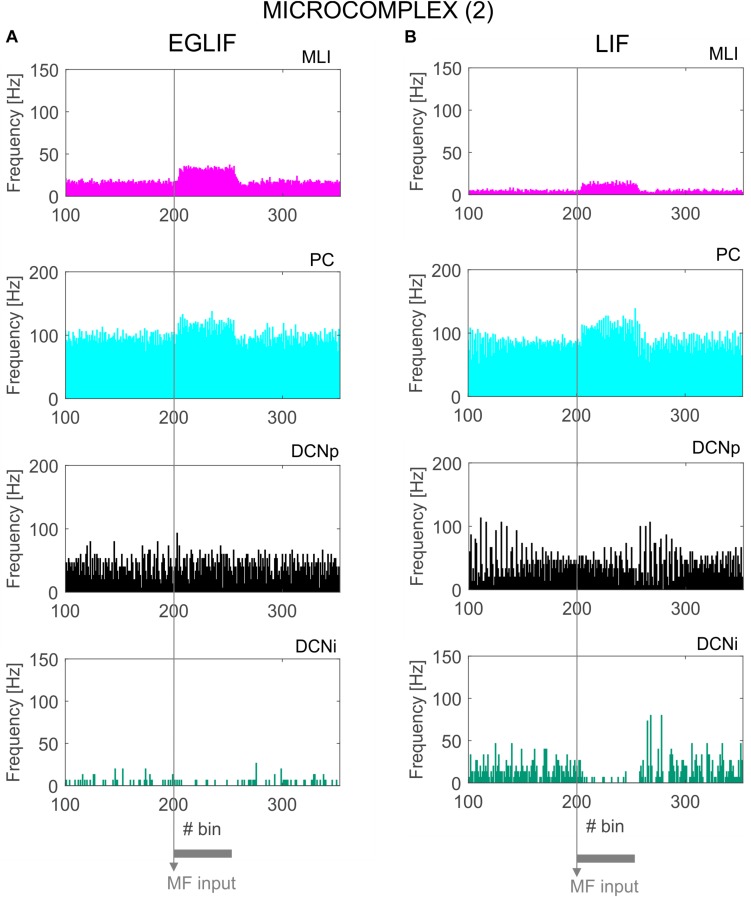
PSTH of MLI, PC and DCN neurons inmicrocomplex (2) in EGLIF-SNN **(A)** and LIF-SNN **(B).** The stimulus causes an increased firing rate in MLIs and PCs. In the nuclear layers, DCNp neurons receive both a higher excitation from MFs and an increased inhibition from PCs due to the stimulus, resulting in a net non-significant change of their firing rate. Conversely, DCNp neurons get silenced by the PCs during the stimulation, as they do not receive MF excitation. Each PSTH bin is 5 ms long.

The basal activity of cerebellar neurons and their response to MF and CF inputs is illustrated in Figures 2–7. In both EGLIF-SNN and LIF-SNN models, during baseline MF activation with random noise at 4 Hz (Rancz et al., 2007), the GrCs were driven into low frequency firing, and the GoC, MLI, PC and DCN neurons slightly increased their firing rate compared to intrinsic pacemaking (Geminiani et al., 2019).

The activity of EGLIF-SNN and LIF-SNN changed during stimulation of the MFs (260 ms at 40 Hz on a MFs bundle, see section “Materials and Methods”) and when a burst was generated in CFs coming from the IO (10 ms at 500 Hz on one microcomplex, see section “Materials and Methods”). At the onset of stimulation, when only MFs were active, the firing rates for all neural populations of the cortical microzones increased with average frequency values within the physiological range. In particular, an increase of about 10 Hz in PC firing rate with respect to 85 Hz baseline emerged, consistent with experimental observations showing that PC activity is largely sustained by pacemaking (Cerminara and Rawson, 2004). The responses of DCN neurons demonstrated a reduction in DCNi, which received only inhibition from PCs, and almost no change in DCNp, which received balanced excitation from MFs and inhibition from PCs, revealing the regulatory power of the system on the cerebellar output. On the other hand, when also the CF burst was injected, complex dynamic spiking patterns were elicited, differentiated in the two microcomplexes; and here the superiority of EGLIF-SNN with respect to LIF-SNN to simulate non-linear responses emerged.

### Granular Layer

Both in EGLIF-SNN and LIF-SNN, the GrCs showed a background low-frequency sparse activation that increased and then recovered to baseline without apparent rebounds. The GoCs also increased firing frequency during the MF stimulus, and then showed a rapid reduction at its end lasting about 30 ms. This was due to slow recovery of the pacemaker cycle reflecting a phase-reset mechanism (Solinas et al., 2007; Geminiani et al., 2018b). The GrCs did not show a corresponding remarkable rebound in their firing rate, probably because of the prolonged effect of GoC-GrC synaptic inhibition, which lasts for about 50 ms (Bengtsson et al., 2013).

### Molecular Layer, PC, and DCN – Microcomplex 1

The activation of IO neurons connected to microcomplex 1 caused a characteristic spiking pattern. In the EGLIF-SNN, the IO input burst induced a typical response in connected PCs, consisting of synchronous complex spikes followed by a pause (*burst-pause*). Each complex spike included a first burst approximating dendritic spikelets, induced by the 10-ms IO input, and a subsequent pause/hyperpolarization, resulting from intrinsic neuron model mechanisms (De Zeeuw et al., 2011; Geminiani et al., 2019). After the burst-pause response, firing recovered but a second firing decrease occurred, caused by spillover-mediated inhibition from MLIs (about 70 ms after the IO burst onset). The PC complex spikes triggered by the IO silenced DCNp neurons (pause), which, after the hyperpolarization, generated a rebound burst. The DCNp *pause-burst* response matches neurophysiological observations (Pugh and Raman, 2006; Zheng and Raman, 2010). DCNi received only PC and IO inputs but not MF excitation, they generated a rebound spike after the strong inhibition from PC complex spikes. In the LIF-SNN, the burst-pause regime of PCs and pause-burst regime of DCN cells did not emerge.

### Molecular Layer, PC, and DCN – Microcomplex 2

Neurons belonging to microcomplex 2 received only the MF stimulus causing a net increase of firing rates in MLI, PC and DCNp neurons, and a pause in DCNi cells not receiving MF excitation.

**FIGURE 8:**
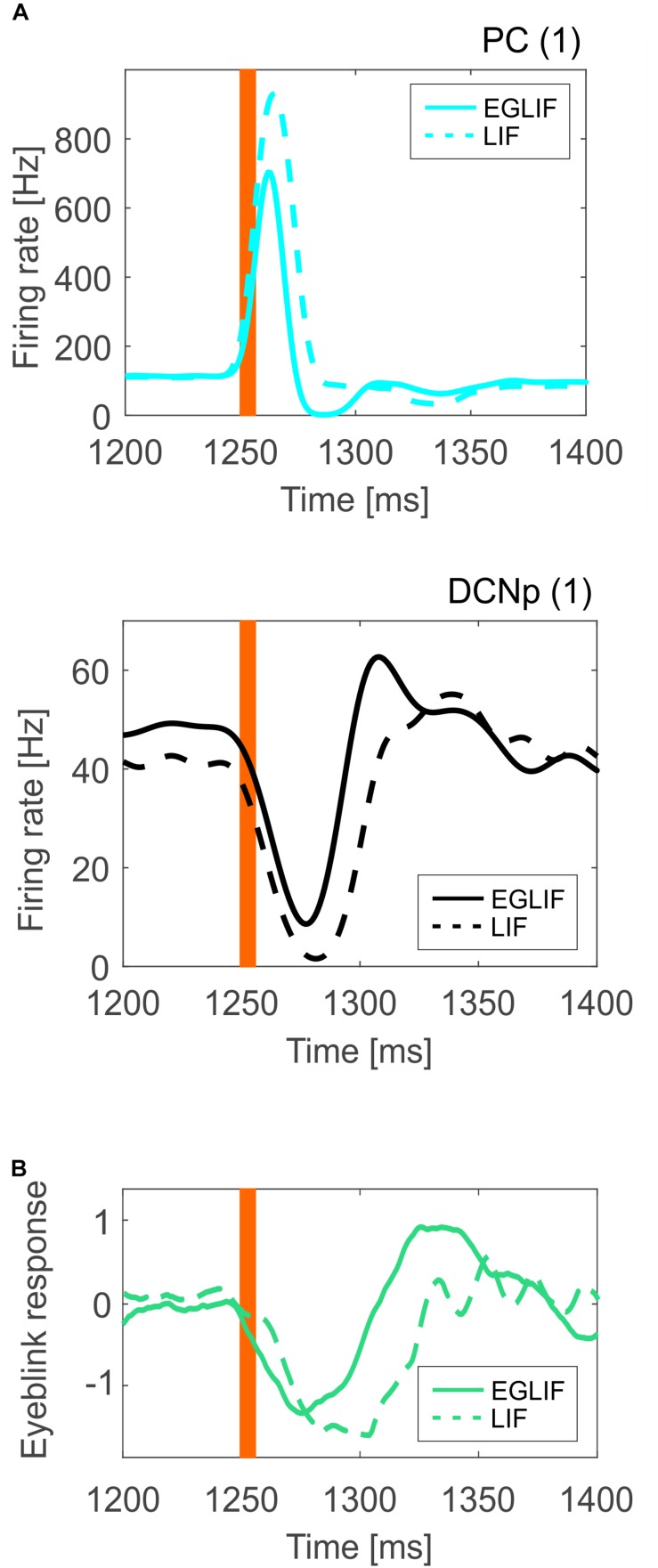
**(A)** Mean instantaneous population firing rate of PC and DCNp neurons from microcomplex (1), averaging all neurons (35 PC and 6 DCNp) and all simulations (*n* = 5), comparing EGLIF-SNN (continuous line) and LIF-SNN (dashed line). The presence of burst-pause and pause-burst responses in EGLIF PC and DCNp neuronal populations, results in a faster and more precise change of the overall population activity (more sensitivity). **(B)** Eyeblink response signal averaged over the five simulations; the DCNp activity of microcomplexes (1) and (2) is first decoded and then the net signal of both microcomplexes is computed to obtain the final response. As a result of the underlying neural mechanisms, the motor response is faster and sharper in the EGLIF-SNN simulations. The orange bar represents the time of the CF bursting input.

For PC and DCNp in the microcomplex 1, where PF and CF stimuli converged, the average firing rate response was sharper in the EGLIF-SNN (Figure 8A), impacting on the timing precision of the network output. Indeed, the dynamic modulation of spike patterns observed using EGLIF could not be reproduced with LIF network models, since the simplified dynamics of single neurons prevented from generating bursting, pause and rebound responses. Consequently, the response *speed* was significantly higher in PC and DCNp neural populations within EGLIF-SNN (PC *speed*: −23.82 ± 1.96 Hz/ms in EGLIF-SNN vs. −2.25 ± 0.91 Hz/ms in LIF-SNN, *t*-test: *p* < 0.01; DCNp *speed*: 1.72 ± 0.83 Hz/ms in EGLIF-SNN vs. 1 ± 0.06 Hz/ms in LIF-SNN, *t*-test: *p* < 0.01).

As a result, the eyeblink response computed from the net decoded activity of DCNp neurons was faster and sharper in the EGLIF-SNN simulations (Figure 8B).

## Discussion

The main observation in this study is that neuron models with realistic non-linear properties EGLIF (Geminiani et al., 2018b, 2019), once embedded into networks with realistic geometry and connectivity (Casali et al., 2019), have a significant impact on ensemble response dynamics compared to simpler models (LIF). The effectiveness of EGLIF emerged as a pattern of burst-pause and pause-burst responses in PC and DCNp neurons reproducing observations *in vivo* (Herzfeld et al., 2015; Moscato et al., 2019) and was most evident when the microcomplex received the CF stimuli. Since we used stimulus patterns emulating those occurring in the eye-blink reflex, it is anticipated that single neuron properties will reverberate on sensorimotor control in closed-loop.

### Single Neuron Activity and SNN Responses to Stimuli

In EGLIF-SNN simulations, the integration of bursts on the CFs and spike trains on PFs proved fundamental for generating a realistic PC output. These stimuli caused PCs to shift from spontaneous background activity to complex spikes and simple spike trains taking the form of a burst-pause response. The burst-pause was the consequence of intrinsic PC non-linear electroresponsive dynamics engaged by patterned synaptic inputs from PFs, MLIs, and IO (Jirenhed et al., 2013). Always in EGLIF-SNN simulations, DCNp neurons showed pause-burst responses deriving from intrinsic DCNp neuron electroresponsiveness engaged by synaptic inputs from PCs, MF and CF collaterals (Herzfeld et al., 2015; Moscato et al., 2019). Indeed, these spiking patterns proved to have a crucial impact on response speed and time precision (Figure 8) providing a potential advantage for cerebellum-driven tasks, in which the cerebellum acts as a millisecond-precise controller (Bareš et al., 2019; Heck et al., 2013). The intrinsic bursting properties of the EGLIF model, already proved in simulations of single neuron responses to current steps (Geminiani et al., 2019), here proved fundamental to capture emergent network dynamics. It should be noted that, in LIF-SNN simulations, burst-pause and pause-burst responses did not emerge. These results therefore support the adequacy of EGLIF neurons for realistic simulations of cerebellar SNNs in closed-loop.

The impact of EGLIF neurons on oscillatory network dynamics, that are expected to emerge from feedback circuit loops in the granular layer (D’Angelo et al., 2013; Maex and De Schutter, 2013), remains to be investigated. Indeed, the intrinsic membrane potential oscillations of EGLIF in single neuron stimulation protocols could impact on network oscillations, and should be further investigated (Geminiani et al., 2019). An open question is also how the EGLIF representation compromises with non-linear dendritic processing in PCs, in which the excitatory post-synaptic potentials are locally amplified by Calcium spikes and integrated into complex spatio-temporal sequences (Masoli et al., 2015; Masoli and D’Angelo, 2017). A similar case applies to DCN cells too, in which the inhibitory post-synaptic potentials set up non-linear interactions with low-threshold calcium spikes (Si Feng et al., 2013). These aspects need to be further investigated by comparison with detailed multicompartmental neuron models.

### Neuronal Wiring and Synaptic Transmission in the SNN

The importance of geometry and connectivity was recently addressed using LIF neurons in a scaffold cerebellar network (Casali et al., 2019). Here the network has been upgraded with EGLIF neurons and extended to include the IO-DCN sub-circuit to form two different microcomplexes, demonstrating additional network properties. In the current configuration, as said, the network generated spiking patterns similar to those observed *in vivo*. A critical issue in this context is the definition of synaptic models (Cavallari et al., 2014). Here we have chosen conductance-based synaptic models implemented with alpha functions, which accounted in an accurate yet simplified form for neurotransmission kinetics (Table 2). A future improvement could be to define conductance changes using specific NMDA, AMPA and GABA kinetics in each neuron type [e.g., see (Wu and Raman, 2017)]. In addition, the more precise spiking patterns of the EGLIF-SNN make this network a better candidate also to investigate short-term plasticity mechanisms. For example, it could be possible to evaluate whether short-term facilitation can further enhance the time precision of the response, amplifying bursting mechanism. In addition, EGLIF-SNN simulations with short-term plasticity could allow to clarify how single neuron and synaptic dynamics interact to generate proper network dynamics.

Finally, phenomena like neurotransmitter spillover and electrical transmission through gap-junctions were approximated here by tuning delay parameters, but could be better reproduced by customized models (Latorre et al., 2013). In GoC and IO neuronal populations, more realistic gap junctions would allow, for instance, to investigate more in detail circuit oscillation properties (Leznik and Llinás, 2005).

### Implications for Eyeblink Conditioning and Other Cerebellum-Driven Paradigms

The stimulation patterns used here mimicked the typical input signals that are used in EBCC tasks including a prolonged and spatially distributed sensory stimulus (CS, light) and a short attentional signal [Unconditioned Stimulus (US), air puff]. The current study focused on the response before learning: CS excited the granular layer across microzones, consistent with the operation of signal analysis (through recombinatorial expansion) carried out by the granular layer (D’Angelo et al., 2013; Gilmer and Person, 2017). The granular layer output was then synthesized and further processed in the PC layer (Dean and Porrill, 2011). US influenced individual microcomplexes through specific IO projections, segregating the attention (or error) signal within the network. These modular activation patterns represent the most elementary instantiation of cerebellar functioning, i.e., the ability to correlate neural signals transmitted along different afferent pathways, the MFs and CFs. These signals, in a behavioral context, are needed to allow the cerebellum *to learn to predict the precise timing of correlated events*, setting the basis for cerebellar contribution to motor and cognitive control (Ivry, 2000; D’Angelo and Casali, 2013). It seems therefore highly relevant that the emerging burst-pause and pause-burst responses in PC and DCNp neurons are precisely reproduced using EGLIF-SNN. These activity patterns will be critical for generating the proper time-locked response in future simulations of EBCC (Rasmussen et al., 2008). This will require to endow the current SNN model with distributed long-term plasticity to simulate learning mechanisms (Antonietti et al., 2016). While the current work evaluated the impact of non-linear single neuron dynamics and network topology on stimulus-response spiking patterns, closed-loop simulations of a full cerebellum-driven learning task with the EGLIF-SNN will allow to evaluate the impact of long-term plasticity, mainly spike-timing dependent plasticity mechanisms, driven by IO and PC spikes.

As a result of modularity and specific connectivity to various brain regions, different cerebellar modules are engaged in different tasks (D’Angelo and Casali, 2013). The modules receive various kinds of input signals, which carry information about specific sensory modalities or specific body parts as well as about activity in motor and associative cortical areas. The modules can differ not only in terms of sources and pathways of the incoming signals, but also in terms of specific electroresponsive properties of neurons. For example, differences in the autorhythm of PCs were observed between regions involved in EBCC and vestibulo-ocular reflexes (Zhou et al., 2014). Similarly, a modulation of oscillatory properties emerge in the IO neural population when encoding either somatosensory or visual stimuli (Llinás, 2014). The possibility to easily modify neuron models and connectivity in our olivocerebellar EGLIF-SNN would allow to fine tune specific features associated to sensorimotor loops and functional cerebellar regions (Casellato et al., 2014; Geminiani et al., 2017; Luque et al., 2019).

According to the modular organization of the cerebellum, these microcomplexes could be multiplied and reconnected to investigate how input signals are integrated and elaborated to control complex movements, for example in whisking and locomotion (Romano et al., 2018). Scaling-up the network modular architecture would require to re-organize connectivity among microcomplexes, which can determine fundamental properties of cerebellar functioning, such as somatotopic organization, fractured somatotopy mapping and multimodal sensory fusion.

## Conclusion

Since the model satisfactorily captures fundamental properties of microcomplexes, it can help shedding light on the links between structure, function and dynamics in the cerebellum under physiological and pathological conditions and during learning (D’Angelo and Gandini Wheeler-Kingshott, 2017). These extended applications are warranted by the flexible structure of the scaffold (Casali et al., 2019) and the tunable nature of EGLIF neurons (Geminiani et al., 2018b, 2019). For example, in different species or in pathological conditions, EGLIF-SNN could account for variations in the number of neurons as well as in their connectivity and intrinsic electroresponsiveness, while maintaining high efficiency when running large-scale simulations in closed-loop. Future work will endow the EGLIF-SNN cerebellum models with mechanisms for synaptic plasticity in order to evaluate the impact of single neuron and network properties on motor learning (Hansel et al., 2001; Schonewille et al., 2010; Gao et al., 2012; D’Angelo, 2014; Boele et al., 2018). Eventually, the model may be exploited to mimic pathological conditions at multiple scales (Geminiani et al., 2018a) providing new insights into the role of cerebellum in brain diseases (D’Angelo and Casali, 2013; D’Angelo, 2019; Schmahmann, 2019). It is also envisaged that the EGLIF scaffold strategy could be customized to model and simulate other brain regions (like the cerebral cortex, hippocampus or basal ganglia).

## Data Availability Statement

All datasets generated for this study are included in the manuscript/Supplementary Files.

## Author Contributions

AG and CC designed and carried out the simulations, performed data analysis and wrote the manuscript. AP, ED’A and CC coordinated the whole work and substantially contributed to the writing of the final manuscript.

## Conflict of Interest

The authors declare that the research was conducted in the absence of any commercial or financial relationships that could be construed as a potential conflict of interest.
