# Supplementary material for: Response Dynamics in an Olivocerebellar Spiking Neural Network With Non-linear Neuron Properties
Source: Front Comput Neurosci. 2019 Oct 1;13:68. doi: 10.3389/fncom.2019.00068 (PMC6779816; doi:10.3389/fncom.2019.00068)
Supplement: Supplementary file 1 [file Table_1.docx]

Response dynamics in an olivocerebellar spiking neural network with non-linear neuron properties

Supplementary material

**Table S1** Single neuron parameters for LIF and EGLIF neuron models

|  | **Common to LIF and EGLIF neurons** | | | | | | **LIF-specific** | **EGLIF-specific** | | | | | | |
| --- | --- | --- | --- | --- | --- | --- | --- | --- | --- | --- | --- | --- | --- | --- |
|  | **C_m_**  [pF] | **τ_m_**  [ms] | **E_L_**  [mV] | **t_ref_**  [ms] | **V_r_**  [mV] | **V_th_**  [mV] | **I_e_**  [pA] | **I_e_**  [pA] | **k_adap_**  [MH^-1^] | **k_2_**  [ms^-1^] | **A_2_**  [pA] | **k_1_**  [ms^-1^] | **A_1_**  [pA] | **λ_0_, τ_V_** |
| ***GoC*** | 145 | 44 | -62 | 2 | -75 | -55 | 36.8 | 16.214 | 0.217 | 0.023 | 178.01 | 0.031 | 259.988 | 1, 0.4 |
| ***GR*** | 7 | 24.15 | -62 | 1.5 | -70 | -41 | 0.0 | -0.888 | 0.022 | 0.041 | -0.94 | 0.311 | 0.01 | 1, 0.3 |
| ***MLI*** | 14.6 | 9.125 | -68 | - | -78 | -53 | 24.05 | 3.711 | 2.025 | 1.096 | 5.863 | 1.887 | 5.953 | 1.8, 1.1 |
| ***PC*** | 334 | 47 | -59 | 0.5 | -69.0 | -43 | 800.0 | 742.534 | 1.491 | 0.041 | 172.622 | 0.195 | 157.622 | 4, 3.5 |
| ***DCNp*** | 142 | 33 | -45 | 1.5 | -55 | -36 | 180.0 | 75.385 | 0.408 | 0.047 | 3.477 | 0.697 | 13.857 | 3.5, 3 |
| ***DCNi*** | 56 | 56 | -40 | 3.02 | -55 | -39 | 40.0 | 2.384 | 0.079 | 0.044 | 176.358 | 0.041 | 176.358 | 0.9, 1 |
| ***IO*** | 189 | 11 | -45 | 1 | -45 | -35 | 0.0 | -18.101 | 1.928 | 0.091 | 1358.197 | 0.191 | 1810.923 | 1.2, 0.8 |

The single neuron parameters are (Geminiani et al., 2018):

$C_{m}$ = membrane capacitance;

$\tau_{m}$ = membrane time constant;

$E_{L}$ = resting potential;

$t_{ref}$ = refractory period;

$V_{r}$ = reset potential;

$V_{th}$ = threshold potential;

$I_{e}$ = endogenous current;

$k_{adap}$, $k_{2}$ = adaptation constants;

$k_{1}$ = decay rate of the intrinsic depolarizing current;

$A_{2}$, $A_{1}$ = model currents update constants;

$\lambda_{0}$, $\tau_{V}$ = escape rate parameters;

All the EGLIF parameters are the same as described in (Geminiani et al., 2019), where an *ad hoc* gradient-based optimization protocol was used to reproduce multiple single neuron spiking patterns (Geminiani et al., 2018). Only the escape rate parameters that allow firing irregularity in the model (i.e. τ_V_ and λ_0_) were increased for PC and DCNp neurons, in order to obtain a higher variability of spike times for *in vivo* network than for *in vitro* single neuron simulations (Boele et al., 2018; Canto et al., 2016). In fact, when moving from single neuron to network simulations, additional noise components that are absent during in vitro experiments have to be taken into account.

The source code of simulations can be found in the following repository: <https://github.com/AliceGem/E-GLIF.git>
